# Plant- and Algae-Derived Compounds Enhance the Anticancer Activity of Doxorubicin in Colorectal Cancer Cell Lines

**DOI:** 10.3390/molecules31142414

**Published:** 2026-07-09

**Authors:** José Alberto Ramos-Silva, Gabriel Lara-Hernández, José Antonio Fuentes-Garibay, Elvia Pérez-Soto, Ericka Patricia Flores-Berrios, Hamlet Avilés-Arnaut

**Affiliations:** 1 Universidad Autónoma de Nuevo León, Facultad de Ciencias Biológicas, Instituto de Biotecnología, Av. Uni-versidad S/N, San Nicolás de los Garza 66455, Nuevo León, Mexico; qfbalbertors@gmail.com (J.A.R.-S.); antonio.fuentesgr@uanl.edu.mx (J.A.F.-G.); 2 Simbio Health Sapi de CV, San José 249, Tlajomulco de Zúñiga 45645, Jalisco, Mexico; gabobmol@gmail.com; 3 Laboratorio de Biomedicina y Salud Ocupacional, Escuela Nacional de Medicina y Homeopatía, Instituto Politécnico Nacional, Mexico City 07320, Mexico; elvperezs@ipn.mx; 4 Investigación y Desarrollo, Biodesarrollos Valmex, Circuito Crisantemos 10, Tlajomulco de Zuñiga 45640, Jalisco, Mexico; eflores@biovalmex.com

**Keywords:** colorectal cancer, doxorubicin, marine algae, apoptosis, caspase-3/7, combined cytotoxic effects, spheroids, natural compounds

## Abstract

Colorectal cancer (CRC) remains one of the leading causes of cancer-related mortality worldwide, and the efficacy of conventional chemotherapy is frequently limited by systemic toxicity, chemoresistance, and tumor recurrence. Natural products derived from marine algae and plants have attracted increasing interest as multitarget adjuvant agents capable of modulating apoptosis, oxidative stress, and tumor-associated signaling pathways. In the present study, we evaluated the anticancer activity of commercially available formulations enriched in fucoxanthin, fucoidan, tocotrienols, astaxanthin, and apple polyphenols, either alone or in combination with doxorubicin (DOX), using two-dimensional and three-dimensional colorectal cancer models. Initial IC_50_ screening in ovarian (OVCAR3), prostate (PC3), colorectal (Caco2 and HT-29), and non-tumorigenic colon epithelial cells demonstrated that formulations 2.1 and 10.0 exhibited the most relevant cytotoxic activity, particularly in colorectal cancer cells. Combined treatments with DOX significantly reduced cell viability compared to individual treatments, particularly in Caco2 cells, where viability decreased to approximately 10% under combined exposure conditions. Mechanistically, combined treatments enhanced caspase-3/7 activation in both Caco2 and HT-29 cells, indicating apoptosis-associated effects. These findings were further supported in three-dimensional spheroid models, where supplement combinations impaired spheroid expansion, induced apoptotic AO/EB staining patterns, and reduced HT-29 spheroid growth by approximately 30–35%, reaching inhibitory effects comparable to DOX alone. Collectively, these results suggest that plant- and algae-derived formulations enriched in antioxidant bioactives may enhance chemotherapy-associated antitumor responses through apoptosis-related mechanisms and modulation of tumor-like growth behavior. The present findings support the further exploration of natural-product-based adjuvant strategies in colorectal cancer therapy using more clinically representative chemotherapeutic schemes and in vivo models.

## 1. Introduction

Colorectal cancer (CRC) remains one of the most significant global health challenges, ranking as the third most diagnosed cancer and the second leading cause of cancer-related death worldwide, according to the Global Cancer Statistics 2020 report [1]. Despite advances in early detection and therapeutic strategies, CRC continues to impose a substantial burden due to late-stage diagnosis, therapeutic resistance, and recurrence [2]. Standard chemotherapeutic strategies for colorectal cancer commonly include fluoropyrimidine-, oxaliplatin-, and irinotecan-based regimens, either alone or in combination with targeted agents [3,4]. However, despite these advances, treatment efficacy may still be limited by systemic toxicity, therapeutic resistance, and tumor recurrence. These limitations highlight the urgent need for alternative or complementary strategies capable of enhancing therapeutic outcomes while minimizing adverse effects [5,6]. Therefore, experimental models using well-characterized cytotoxic agents remain useful to explore whether natural-product-based formulations can modulate chemotherapy-associated cellular responses. In this context, doxorubicin (DOX) was selected in the present study as a reference cytotoxic agent because its mechanisms of action, including DNA damage, topoisomerase II inhibition, oxidative stress, and apoptosis induction, have been extensively characterized in cancer models [7,8,9,10]. Thus, DOX was used here as a proof-of-concept chemotherapeutic stressor rather than as a standard clinical backbone for colorectal cancer therapy.

Among emerging approaches, plant- and marine-derived bioactive compounds have attracted considerable attention due to their antioxidant, anti-inflammatory, and anticancer properties [11,12]. Compounds such as fucoxanthin, fucoidan, plant-derived tocotrienols, and phenolic compounds have demonstrated the ability to modulate key cellular processes involved in tumor progression, including proliferation, apoptosis, oxidative stress, and signaling pathways associated with survival and metastasis [13,14,15,16]. Previous studies have shown that these compounds can induce cytotoxic and pro-apoptotic effects in colorectal cancer cell lines such as Caco2 and HT-29, often through mechanisms involving mitochondrial dysfunction, modulation of Bcl-2 family proteins, and activation of caspase-dependent pathways [17,18,19]. Additionally, growing evidence suggests that such natural compounds may exhibit adjuvant-like activity when combined with conventional chemotherapeutic agents, potentially contributing to enhanced antitumor responses [20,21].

Despite this growing body of evidence, a critical gap remains between the study of isolated bioactive compounds and the evaluation of complex, commercially available formulations that contain multiple active ingredients. Limited information exists regarding the combined effects of such formulations with standard chemotherapeutic drugs in biologically relevant models, including three-dimensional (3D) systems that better recapitulate tumor architecture, cell–cell interactions, and drug diffusion dynamics [22,23,24]. Therefore, the potential of plant- and algae-derived mixtures as adjuvant agents in colorectal cancer therapy remains insufficiently explored.

To address this need, the present study aimed to evaluate the cytotoxic, pro-apoptotic, and functional effects of selected plant- and algae-derived dietary supplements, both individually and in combination with doxorubicin, across multiple cancer models, with a particular focus on colorectal cancer cell lines (Caco2 and HT-29). The study integrates two-dimensional viability assays, caspase-3/7 activation analysis, and three-dimensional spheroid models to determine whether these dietary supplements can produce enhanced cytotoxic and apoptosis-associated responses when combined with a reference chemotherapeutic stressor.

## 2. Results

### 2.1. Cytotoxic Activity of Plant- and Algae-Derived Supplements in Cancer Cell Lines

The initial aim of this study was to determine whether the evaluated plant- and algae-derived supplements exhibited intrinsic cytotoxic activity that could support their subsequent evaluation as adjuvants in chemotherapy. IC_50_ values were determined after 24 h of treatment using the MTT assay in ovarian (OVCAR3), prostate (PC3), colorectal (Caco2 and HT-29), and non-tumorigenic colon epithelial (CCD-481) cell lines (Table 1).

Supplements 2.0, 3.0, and 4.0 showed IC_50_ values higher than 1000 µg/mL in all evaluated cell lines, indicating limited cytotoxic activity under the tested conditions. Therefore, subsequent analyses focused exclusively on the formulations displaying the highest biological activity, namely supplements 2.1 and 10.0.

Overall, supplement 10.0 exhibited cytotoxic activity across all tumor cell lines evaluated, although with variable potency depending on the cancer model. The lowest sensitivity to supplement 10.0 was observed in the ovarian cancer cell line OVCAR3, which displayed an IC_50_ value of 783.7 ± 58.43 µg/mL. This reduced sensitivity may be associated with the intrinsic multidrug-resistant phenotype of OVCAR3 cells, as this line was originally established from a patient previously treated with cyclophosphamide, doxorubicin, and cisplatin, which contributed to the selection of chemoresistant tumor cells [25]. In contrast, PC3 prostate cancer cells showed moderate sensitivity to supplement 10.0, with an IC_50_ value of 156.5 ± 34.5 µg/mL.

Interestingly, colorectal cancer cells were among the most sensitive models to supplement 10.0. In Caco2 cells, the IC_50_ value was 158.70 ± 17.79 µg/mL, whereas HT-29 cells exhibited markedly higher sensitivity, with an IC_50_ value of 28.6 ± 6.08 µg/mL. Similarly, supplement 2.1 displayed a preferential effect toward colorectal cancer cells. While moderate cytotoxicity was observed in Caco2 cells (IC_50_ = 340.50 ± 59.13 µg/mL), the effect was considerably more potent in HT-29 cells, where the IC_50_ reached 90.99 ± 28.09 µg/mL. Importantly, the non-tumorigenic CCD-481 colon epithelial cells were minimally affected by supplement 2.1, showing IC_50_ values greater than 1000 µg/mL, suggesting a degree of selectivity toward tumor cells. In contrast, formulation 10.0 displayed measurable activity in CCD-481 cells, suggesting a lower degree of selectivity than formulation 2.1. This observation underscores the importance of further safety evaluations before considering the formulation for translational applications. To further evaluate the relative tumor selectivity of the most active treatments, selectivity index (SI) values were calculated using CCD-481 cells as the non-tumorigenic reference model. Formulation 2.1 showed the most favorable selectivity profile, with SI values of >2.94 for Caco2 cells and >10.99 for HT-29 cells. In contrast, formulation 10.0 displayed lower selectivity, with SI values of 0.26 for Caco2 cells and 1.43 for HT-29 cells, reflecting its measurable cytotoxic activity in CCD-481 cells. DOX showed SI values of 6.05 and 4.13 for Caco2 and HT-29 cells, respectively. These findings suggest that formulation 2.1 may possess a more favorable in vitro therapeutic window, although additional studies are required to confirm its safety under combination-treatment conditions.

It is important to note that doxorubicin displayed substantially higher potency than the evaluated dietary supplements in all cell lines tested, with IC_50_ values ranging from 6.50 to 39.3 µg/mL. This difference is expected because doxorubicin is a clinically optimized antineoplastic drug specifically designed to target rapidly proliferating cancer cells, whereas the evaluated supplements correspond to complex mixtures of natural bioactive compounds, which generally exhibit lower intrinsic potency but may still provide relevant biological effects and potential adjuvant properties.

Collectively, these findings indicate that certain formulations (particularly 10.0 and, to a lesser extent, 2.1) exhibit biologically relevant activity, with a more pronounced effect in colorectal cancer models, thereby supporting their further evaluation in combination with doxorubicin.

### 2.2. Effects of Combined Treatments with Supplements and Doxorubicin in Colon Cancer Cells

Based on the IC_50_ results (Table 1), which showed that formulations 10.0 and 2.1 exhibited the most relevant cytotoxic activity in colorectal cancer cells, and considering the high global incidence of colorectal cancer, subsequent experiments were conducted exclusively using Caco2 and HT-29 models to evaluate the effects of combined treatments with doxorubicin (DOX).

**Figure 1:**
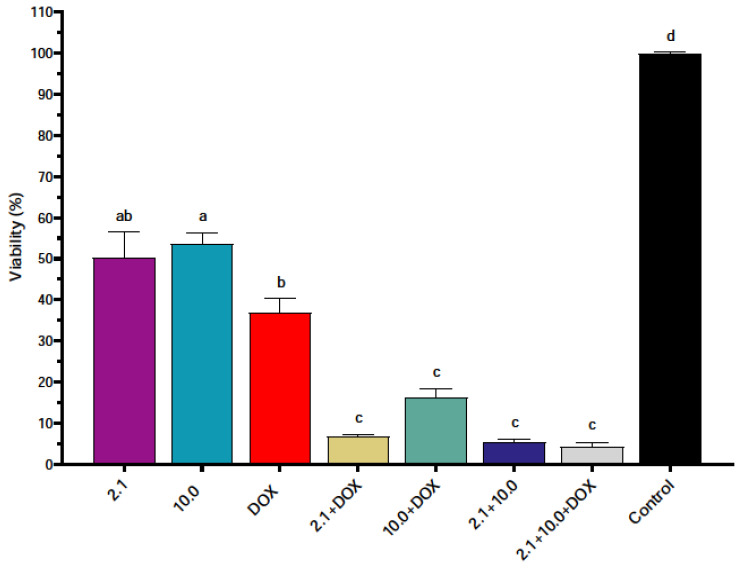
Effects of plant- and algae-derived supplements combined with doxorubicin on Caco2 cell viability. Effects of individual and combined treatments with supplements 2.1 and 10.0 and doxorubicin (DOX) on Caco2 cell viability after 24 h. Each bar represents the mean ± SEM (*n* = 3). Treatments were applied at the IC_50_ concentration of each compound. The control group consisted of untreated cells. Statistical analysis was performed using one-way ANOVA followed by Tukey’s post hoc test. Different letters indicate statistically significant differences between groups (*p* < 0.05).

In Caco2 cells, combinations of supplements with DOX produced a marked reduction in cell viability compared to the individual treatments, which by definition were close to 50% viability at their respective IC_50_ concentrations (Figure 1). Notably, when supplement 2.1 was combined with DOX, cell viability decreased to approximately 10%, indicating that the combined treatment exerted a substantially stronger inhibitory effect than either treatment alone. Similarly, the combination of supplement 10.0 with DOX further reduced viability relative to single-agent exposure. Interestingly, the combination of supplements 2.1 + 10.0 produced an even greater reduction in viability than treatment with DOX alone, suggesting that the mixture of natural bioactive compounds itself can strongly affect colorectal cancer cell survival.

**Figure 2:**
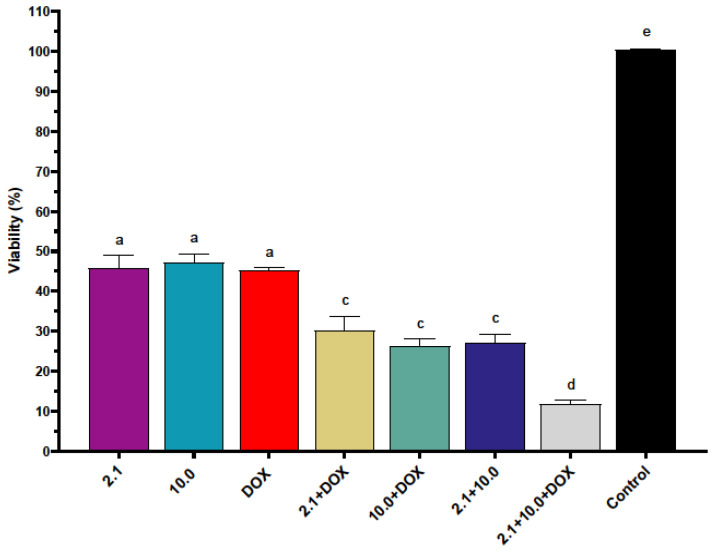
Effects of plant- and algae-derived supplements combined with doxorubicin on HT-29 cell viability. Effects of individual and combined treatments with supplements 2.1 and 10.0 and doxorubicin (DOX) on HT-29 cell viability after 24 h. Each bar represents the mean ± SEM (*n* = 3). Treatments were applied at the IC_50_ concentration of each compound. The control group consisted of untreated cells. Statistical analysis was performed using one-way ANOVA followed by Tukey’s post hoc test. Different letters indicate statistically significant differences between groups (*p* < 0.05).

A similar pattern was observed in HT-29 cells (Figure 2), where combined treatments resulted in a marked decrease in viability relative to single-agent exposure. However, the double combinations appeared less potent than those observed in Caco2 cells, as viability reductions generally remained above the levels reached in the Caco2 model. In HT-29 cells, only the triple treatment containing DOX, 2.1, and 10.0 reduced viability to levels close to 10%, representing the strongest inhibitory effect observed in this cell line. Together, these findings indicate that selected formulations not only display intrinsic cytotoxic activity but also enhance the effectiveness of doxorubicin in colorectal cancer cells, particularly when multiple bioactive formulations are combined, supporting their further evaluation in caspase-mediated apoptotic pathways, which are explored in the following section.

### 2.3. Caspase Activation in Caco2 Cells

To explore whether the observed reduction in viability was associated with apoptotic mechanisms, caspase 3/7 activation was evaluated in Caco2 cells.

**Figure 3:**
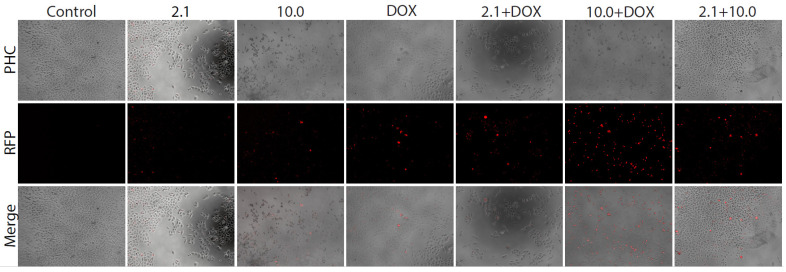
Fluorescence microscopy analysis of caspase 3/7 activation in Caco2 cells. Representative phase contrast (PHC), RFP fluorescence, and merged images showing caspase 3/7 activation in Caco2 cells after treatment with supplements 2.1, 10.0, doxorubicin (DOX), and their combinations. Increased red fluorescence indicates activation of caspase-dependent apoptotic pathways. Each image represents a typical outcome from three separate experiments.

Fluorescence microscopy analysis revealed an increased presence of caspase-positive cells in treatments involving doxorubicin and its combinations with the evaluated supplements. While control cells displayed minimal basal fluorescence, indicative of low apoptotic activity, treatments with supplements alone showed only a modest increase in caspase-positive signals. In contrast, cells treated with DOX, particularly in combination with supplement 10.0, exhibited a marked rise in red fluorescence, consistent with enhanced caspase 3/7 activation (Figure 3).

**Figure 4:**
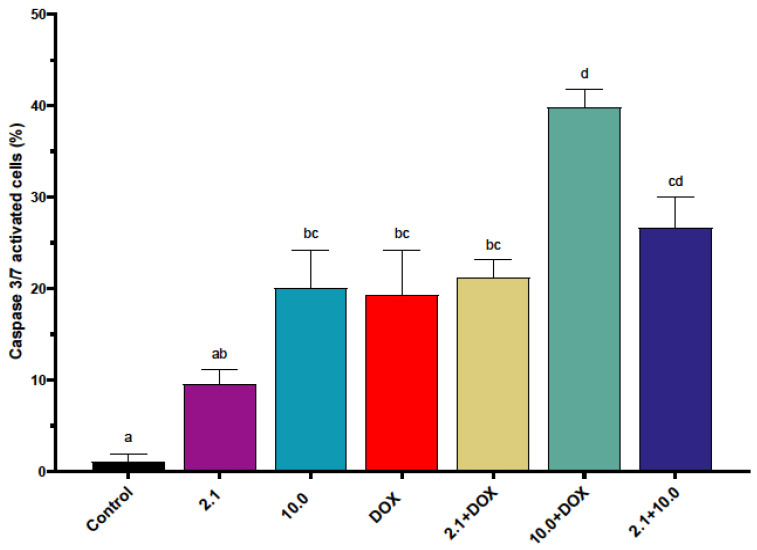
Quantification of caspase 3/7-positive Caco2 cells following treatment. Percentage of caspase 3/7-positive Caco2 cells after treatment with supplements 2.1, 10.0, DOX, and their combinations. Each bar represents mean ± SEM (*n* = 3). Treatments were applied at the IC_50_ concentration of each compound. Control corresponds to untreated cells. Statistical analysis was performed using one-way ANOVA followed by Tukey’s post hoc test. Different letters indicate statistically significant differences (*p* < 0.05).

The proportion of caspase-positive cells was significantly elevated in treatments combining supplements with doxorubicin compared to the control and single-agent treatments. Notably, the 10.0 + DOX combination produced the highest percentage of caspase 3/7-positive cells, reaching approximately 40%, whereas treatment with DOX alone induced caspase activation in approximately 20% of the cells, indicating enhanced apoptotic signaling under combined exposure conditions (Figure 4). In contrast, the treatment showing the lowest caspase activity corresponded to supplement 2.1 alone, which induced caspase activation in only approximately 10% of the cells.

Interestingly, treatments with supplement 10.0 alone and DOX alone did not show statistically significant differences in caspase activation levels in Caco2 cells, despite both treatments producing higher apoptotic activity than the untreated control. These findings suggest that the enhanced apoptotic response observed under combined treatments may result from the interaction of multiple bioactive compounds with doxorubicin-induced stress pathways.

Together, these findings suggest that the enhanced reduction in viability observed in Caco2 cells under combined exposure conditions may be mediated, at least in part, through activation of caspase-dependent apoptotic pathways. These results provide mechanistic support for the enhanced apoptotic response observed following the combined treatment of plant- and algae-derived compounds with doxorubicin in colorectal cancer cells.

### 2.4. Caspase Activation in HT-29 Cells

**Figure 5:**
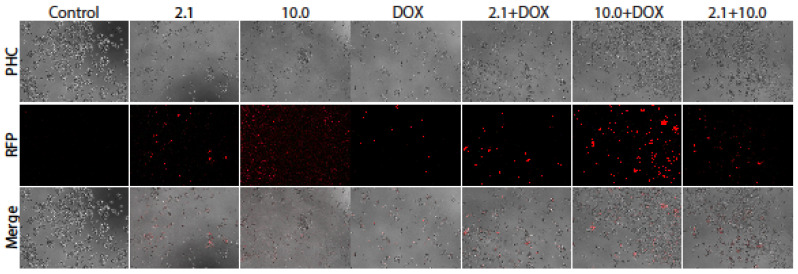
Fluorescence microscopy analysis of caspase 3/7 activation in HT-29 cells. Representative phase contrast (PHC), RFP fluorescence, and merged images showing caspase 3/7 activation in HT-29 cells after treatment with supplements 2.1, 10.0, doxorubicin (DOX), and their combinations. Increased red fluorescence indicates activation of apoptotic pathways. Each image represents a typical outcome from three separate experiments.

**Figure 6:**
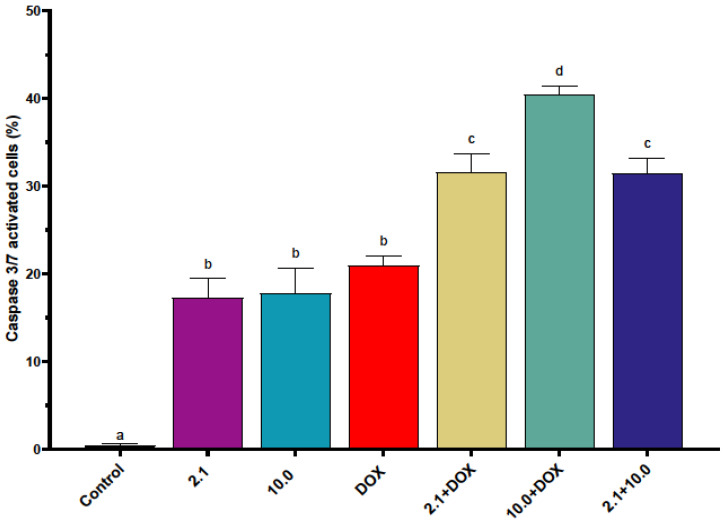
Quantification of caspase 3/7-positive HT-29 cells following treatment. Percentage of caspase 3/7-positive HT-29 cells after treatment with supplements 2.1, 10.0, DOX, and their combinations. Each bar represents mean ± SEM (*n* = 3). Treatments were applied at the IC_50_ concentration of each compound. Control corresponds to untreated cells. Statistical analysis was performed using one-way ANOVA followed by Tukey’s post hoc test. Different letters indicate statistically significant differences (*p* < 0.05).

Caspase 3/7 activation was also evaluated in HT-29 cells to determine whether the reduction in viability observed under combined treatments was associated with apoptotic signaling. Fluorescence microscopy revealed an increase in caspase-positive cells following treatment with doxorubicin and its combinations with the evaluated supplements. Control cells exhibited minimal red fluorescence, indicating low basal caspase activity. Treatments with supplements alone produced a modest increase in caspase activation; however, treatments containing doxorubicin, particularly the 10.0 + DOX combination, displayed the highest fluorescence signal, indicating increased activation of apoptotic pathways (Figure 5). These observations were confirmed by semi-quantitative analysis (Figure 6). Similar to the findings obtained in Caco2 cells, the treatment 10.0 + DOX produced the highest percentage of caspase 3/7-positive cells, reaching approximately 40%, followed by 2.1 + DOX (with 30%), whereas treatment with DOX alone induced caspase activation in approximately 20% of the cells. These results indicate an enhanced apoptotic response under combined exposure conditions.

In contrast to the behavior observed in Caco2 cells, treatment with supplement 2.1 alone in HT-29 cells did not show significant differences compared to treatment with DOX alone in terms of the percentage of caspase-positive cells. This observation suggests that HT-29 cells may be intrinsically more sensitive to one or more bioactive compounds present in supplement 2.1, potentially contributing to the higher cytotoxic response previously observed in this cell line (Figure 6).

Together with the findings in Caco2 cells, these results indicate that the reduction in viability observed in colorectal cancer models is associated, at least in part, with activation of caspase-dependent apoptotic pathways. These findings further support the association between the observed reduction in cell viability and the activation of caspase-dependent apoptotic pathways following combined treatment with plant- and algae-derived formulations and doxorubicin.

### 2.5. Evaluation of Cytotoxic Effects in a 3D Spheroid Model

Although 2D models are widely used to evaluate cytotoxic responses, they do not always recapitulate the structural and biological complexity of solid tumors. To improve the simulation of the potential effects of the evaluated supplements on colorectal tumors, cytotoxicity was further assessed using a three-dimensional spheroid model.

In addition to monitoring spheroid growth—expressed as diameter over time up to day 5—apoptosis was evaluated through dual fluorescent staining with acridine orange and ethidium bromide (AO/EB). This method allowed the visualization of viable cells (green), early and late apoptotic cells (yellow and orange), and necrotic cells (red).

**Figure 7:**
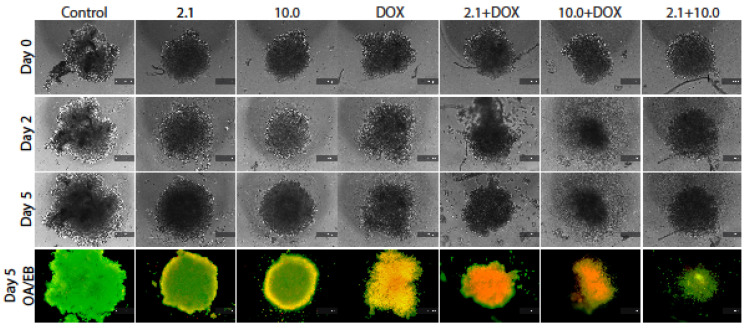
Effects of supplements and doxorubicin on Caco2 spheroid morphology and apoptosis. Representative images of Caco2 spheroids treated with supplements 2.1, 10.0, doxorubicin (DOX), and their combinations at days 0, 2, and 5. Dual staining with acridine orange/ethidium bromide (AO/EB) was performed on day 5 to evaluate cell viability and death. Green fluorescence indicates viable cells, yellow and orange correspond to early and late apoptosis, and red fluorescence indicates necrosis. Scale bars represent 100 μm in all micrographs.

While control spheroids displayed continued expansion over time, treatments containing doxorubicin, particularly in combination with supplements, led to more compact or disrupted spheroid structures by day 5. AO/EB staining further showed increased apoptotic signals in combined treatments, evidenced by the presence of yellow and orange fluorescence, whereas control spheroids predominantly exhibited green fluorescence consistent with viable cells (Figure 7).

**Figure 8:**
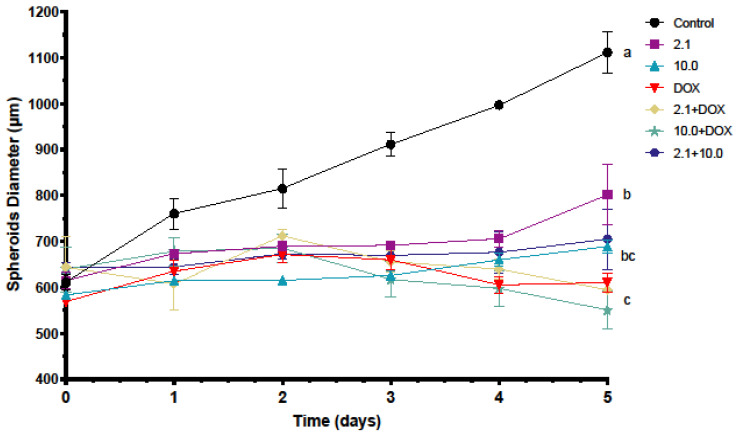
Growth kinetics of Caco2 spheroids under individual and combined treatments. Time-course analysis of Caco2 spheroid diameter over 5 days following treatment with supplements 2.1, 10.0, DOX, and their combinations. Each point represents mean ± SEM (*n* = 3). Statistical analysis was performed using one-way ANOVA followed by Tukey’s post hoc test. Different letters indicate statistically significant differences (*p* < 0.05).

Control spheroids demonstrated continuous growth, reaching the largest diameter by day 5. In contrast, treatments combining doxorubicin with supplements significantly limited spheroid expansion compared to untreated controls and single-agent treatments. Among the evaluated conditions, DOX + 10.0 showed the most pronounced reduction in spheroid size (Figure 8). Together, these findings indicate that the combined treatments not only reduce cell viability in 2D cultures but also impair spheroid growth and are associated with increased apoptosis-related responses in a 3D tumor-like environment. Collectively, these observations support the potential use of plant- and algae-derived formulations as adjuvant agents capable of enhancing the cytotoxic effects of doxorubicin in colorectal cancer models.

### 2.6. Evaluation of Cytotoxic Effects in HT-29 3D Spheroids

**Figure 9:**
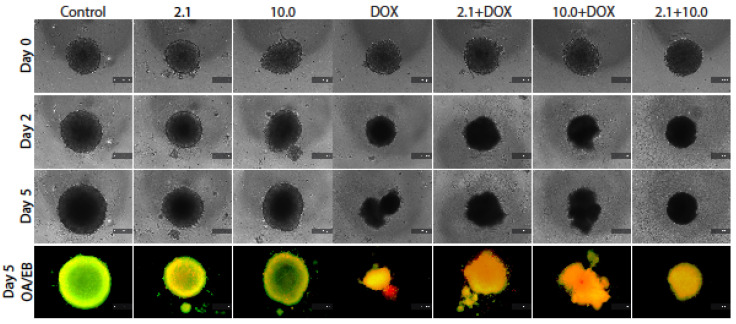
Effects of supplements and doxorubicin on HT-29 spheroid morphology and apoptosis. Representative images of HT-29 spheroids treated with supplements 2.1, 10.0, doxorubicin (DOX), and their combinations at days 0, 2, and 5. Dual staining with acridine orange/ethidium bromide (AO/EB) was performed on day 5 to evaluate cell viability and death. Green fluorescence indicates viable cells, yellow and orange correspond to early and late apoptosis, and red fluorescence indicates necrosis. Scale bars represent 100 μm in all micrographs.

To further validate the functional impact of the evaluated treatments in a more physiologically relevant context, cytotoxicity was assessed in HT-29 three-dimensional spheroids. As observed in Caco2, morphological analysis revealed progressive differences in spheroid integrity across treatments. While control spheroids maintained structural integrity and continued growth over time, treatments involving doxorubicin, particularly in combination with supplements, resulted in smaller and less compact spheroids by day 5. Dual acridine orange/ethidium bromide (AO/EB) staining confirmed increased apoptotic signals in combined treatments, evidenced by the presence of yellow and orange fluorescence, whereas control spheroids predominantly exhibited green fluorescence corresponding to viable cells (Figure 9).

**Figure 10:**
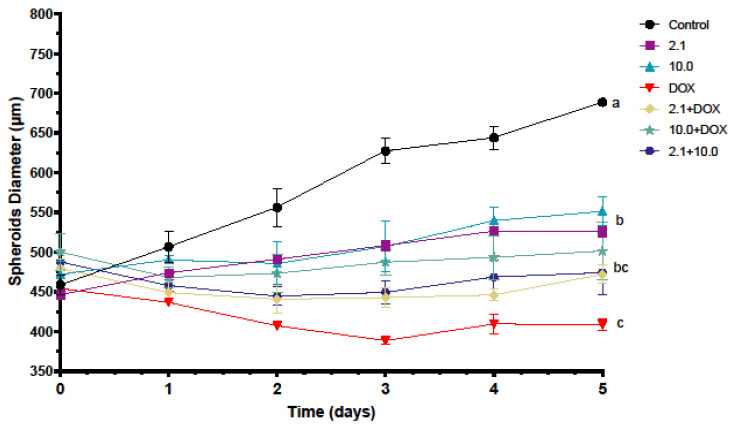
Growth kinetics of HT-29 spheroids under individual and combined treatments. Time-course analysis of HT-29 spheroid diameter over 5 days following treatment with supplements 2.1, 10.0, DOX, and their combinations. Each point represents mean ± SEM (*n* = 3). Statistical analysis was performed using one-way ANOVA followed by Tukey’s post hoc test. Different letters indicate statistically significant differences (*p* < 0.05).

Control spheroids showed continuous growth, reaching approximately 690–700 µm in diameter by day 5. In contrast, spheroids treated with the combination of supplements 2.1 + 10.0 reached only ~460 µm at the same time point, representing an approximate reduction of ~30–35% in size compared to untreated controls. Importantly, this reduction was comparable to that observed in the DOX-treated group, and no significant difference was detected between the 2.1 + 10.0 combination and doxorubicin alone, as indicated by shared statistical grouping (Figure 10).

Together, these results demonstrate that the supplement combination can achieve a growth-inhibitory effect in 3D tumor-like structures similar to that of doxorubicin, further supporting the potential of the supplements in modulating tumor behavior in more complex models.

## 3. Discussion

This study demonstrates that selected natural supplements (formulations 2.1 and 10.0) not only display intrinsic cytotoxicity in ovary, prostate and colorectal cancer cells but also enhance the anticancer activity of doxorubicin. These effects are supported by increased caspase-3/7 activation, inhibition of tumor growth in three-dimensional spheroid models, and the promotion of apoptosis. The decision to focus on Caco2 and HT-29 was supported by the IC_50_ profile (Table 1), where these colon models showed the most pronounced responsiveness to formulations 2.1 and especially 10.0, relative to other tumor types.

A noteworthy finding of the present study was the measurable cytotoxic activity of formulation 10.0 in the non-tumorigenic CCD-481 colon epithelial cell line. Although the IC_50_ value obtained for CCD-481 cells (40.98 ± 4.6 μg/mL) was higher than that observed in HT-29 colorectal cancer cells (28.6 ± 6.08 μg/mL), suggesting a preferential effect toward this tumor model, it was substantially lower than the IC_50_ determined for Caco2 cells (158.7 ± 12.5 μg/mL). These findings indicate that the biological activity of formulation 10.0 varies among different colorectal cancer cell models and suggest that some bioactive constituents present in this formulation may exert biological effects that are not entirely restricted to malignant cells. One possible explanation is the relatively high concentration and broad spectrum of bioactive compounds present in formulation 10.0, including fucoxanthin, fucoidan, tocotrienols, astaxanthin, and other antioxidant phytochemicals. While these compounds have been widely reported to induce apoptosis, inhibit proliferation, and modulate oxidative stress in cancer cells, several studies have shown that at sufficiently high concentrations they may also affect normal proliferating cells by altering redox homeostasis, mitochondrial function, or cell-cycle progression [26,27,28,29,30]. Therefore, the biological activity observed in CCD-481 cells may reflect the multitarget nature of these compounds rather than a strictly tumor-specific mechanism.

From a translational perspective, these findings highlight the importance of carefully evaluating therapeutic windows and selectivity profiles when considering complex natural-product formulations as adjuvant agents. Although the present results suggest a preferential effect toward colorectal cancer cells, additional studies involving expanded panels of non-tumorigenic cells, long-term exposure experiments, and in vivo toxicity assessments will be necessary to establish the safety profile of formulation 10.0 and determine whether its potential therapeutic benefits outweigh possible off-target effects. Interestingly, formulation 10.0 exhibited measurable cytotoxic activity in the non-tumorigenic CCD-481 colon epithelial cell line. This was further reflected in the selectivity index analysis, where formulation 10.0 showed SI values of 0.26 for Caco2 and 1.43 for HT-29, indicating limited tumor selectivity, particularly in Caco2 cells. In contrast, formulation 2.1 exhibited a more favorable selectivity profile, with SI values of >2.94 for Caco2 and >10.99 for HT-29. These findings suggest that formulation 2.1 may have a wider in vitro selectivity window, whereas formulation 10.0 requires additional safety evaluation before being considered for translational applications.

The SI values obtained for formulation 2.1 are comparable to or higher than those reported for several plant-derived bioactive compounds evaluated in colorectal cancer models. For example, an active fraction from *Physalis angulata* calyces exhibited SI values of 1.52 in Caco-2 cells and 3.24 in HT-29 cells when compared with CCD 841 CoN normal colon epithelial cells [31]. Similarly, the flavonol isorhamnetin showed an SI value of 2.69 in HT-29 cells, while cedrol, a sesquiterpene alcohol isolated from plant sources, displayed SI values ranging from 1.46 to 3.05 in colorectal cancer models [32,33]. Therefore, the high selectivity observed for formulation 2.1, particularly in HT-29 cells, supports its potential as a candidate for further investigation. Nevertheless, because the combination treatments were not evaluated in non-tumorigenic cells, additional studies will be required to determine whether tumor selectivity is maintained under combination-treatment conditions.

The translational relevance of these findings should be interpreted with caution, as DOX was used as the chemotherapeutic agent in colorectal cancer models, whereas fluoropyrimidine-, oxaliplatin-, and irinotecan-based regimens are more clinically representative [3,4]. Nevertheless, DOX was selected as a well-characterized reference cytotoxic agent to establish a proof-of-concept model for evaluating the effects of plant- and algae-derived formulations on chemotherapy-associated responses [7,8,9,10]. Therefore, future studies should assess these formulations in combination with 5-fluorouracil, oxaliplatin, and irinotecan to determine their translational relevance in colorectal cancer therapy.

A key finding is that co-treatment of supplements with doxorubicin increased cytotoxicity in both Caco2 and HT-29, consistent with previous evidence that macroalgae-derived bioactives can sensitize colorectal cancer cells to stress and apoptotic stimuli [34]. The observed effects are likely mediated by the complex and multitarget nature of supplements, which are rich in antioxidants such as astaxanthin, fucoxanthin, fucoidan, polyphenols and tocotrienols. For example, fucoxanthin, one of the most studied brown-algae carotenoids, reduces viability and induces apoptosis in colon cancer models including Caco2 and HT-29, through caspase-dependent pathways [35,36]. Likewise, fucoidan, a sulfated polysaccharide from brown algae, has been repeatedly reported to suppress HT-29 growth and to promote apoptotic signaling, including activation of caspase cascades, mitochondrial membrane permeability and suppressing pro-survival signaling pathways, such as PI3K/Akt/mTOR [37,38,39], aligning with our observation about supplement–DOX combinations increased caspase-3/7-positive cells.

Astaxanthin, a xanthophyll carotenoid present in marine organisms and microalgae such as *Haematococcus pluvialis*, has demonstrated important antitumoral activity in colorectal cancer models, including HT-29, HCT116, and other colon cancer cell lines. In HT-29 colorectal cancer cells, astaxanthin increased caspase-3 activation and downregulated EGFR/HER2/ERK signaling, supporting its role as a pro-apoptotic compound capable of interfering with survival pathways commonly associated with chemoresistance [40]. Although astaxanthin has been reported to modulate oxidative stress and redox-sensitive signaling pathways in colorectal cancer models, these mechanisms were not directly evaluated in the present study and therefore should be considered as potential contributors requiring future investigation.

Tocotrienols, members of the vitamin E family commonly found in palm oil, rice bran, and annatto, have demonstrated significant antitumoral activity in colorectal cancer models through multiple molecular mechanisms associated with apoptosis, cell cycle arrest, and modulation of inflammatory signaling pathways. Several studies have reported that γ- and δ-tocotrienols inhibit the proliferation of HT-29, Caco-2, HCT116, and DLD-1 colorectal cancer cells while promoting caspase-dependent apoptosis and suppression of survival signaling. In particular, δ-tocotrienol was shown to suppress colorectal tumorigenesis both in vitro and in vivo by activating caspase-3 and caspase-9 while simultaneously inhibiting the COX-2/PGE2 pathway involved in tumor–stromal interactions and inflammatory signaling within the tumor microenvironment [41]. Additional reports demonstrated that tocotrienols can induce G0/G1 cell-cycle arrest, increase expression of cyclin-dependent kinase inhibitors such as p21 and p27, and interfere with pathways including NF-κB, PI3K/Akt, VEGF, and HIF-1α, all of which are closely associated with proliferation, angiogenesis, and chemoresistance in colorectal cancer [42]. Furthermore, γ- and δ-tocotrienols have been reported to enhance apoptotic responses and inhibit spheroid growth in colorectal cancer models, supporting their potential relevance as multitarget adjuvant compounds capable of modulating both tumor cells and the surrounding microenvironment [43].

Apple polyphenols have demonstrated significant antitumoral activity in colorectal cancer models through mechanisms involving apoptosis induction, inhibition of proliferation, modulation of oxidative stress, and interference with glucose metabolism and survival signaling pathways. Several studies using HT-29 and Caco-2 cells have shown that apple-derived polyphenolic compounds, particularly phloretin, procyanidins, catechins, and chlorogenic acid derivatives, can reduce cell viability and promote apoptotic cell death in colon cancer cells. Phloretin has been reported to inhibit colorectal cancer growth through suppression of GLUT2-mediated glucose uptake and activation of p53-associated signaling pathways, thereby impairing tumor cell metabolism and survival [44]. Likewise, apple flavonoids and procyanidin-rich extracts have shown antiproliferative effects in HT-29 cells associated with apoptosis induction and modulation of oxidative stress-related pathways [45]. In addition, studies have demonstrated that these compounds can regulate ROS production, antioxidant enzymes, DNA fragmentation, and caspase-3 activation, supporting their role as multitarget bioactive agents capable of modulating redox balance and apoptotic signaling in colorectal cancer cells [46]. Collectively, these findings support the hypothesis that the apple polyphenols present in formulation 2.1 may contribute to the enhanced antitumoral activity observed in the present study through mechanisms previously associated with metabolic disruption and apoptosis induction. However, because oxidative stress-related parameters were not directly evaluated, the contribution of redox-regulatory pathways remains to be determined.

The differential response observed between Caco-2 and HT-29 cells suggests that the biological characteristics of each colorectal cancer model may influence their sensitivity to the supplements and their combinations with doxorubicin. Caco-2 cells exhibit a more differentiated enterocyte-like phenotype, whereas HT-29 cells are generally considered less differentiated and more heterogeneous, with important differences in metabolism, mucin production, and signaling pathways associated with chemoresistance [47,48].

Beyond their differences in differentiation status, Caco-2 and HT-29 cells also exhibit distinct molecular characteristics that may contribute to the differential responses observed in the present study. Caco-2 cells express higher levels of ATP-binding cassette (ABC) transporters associated with increased resistance to several anticancer agents, including paclitaxel, vinblastine, irinotecan, and etoposide, whereas HT-29 cells exhibited greater sensitivity to formulations 2.1 and 10.0. Together with differences in signaling pathways such as PI3K/Akt and MAPK/ERK, these characteristics may contribute to the distinct responses observed between both colorectal cancer cell lines [49,50,51].

These intrinsic differences could partially explain the distinct apoptotic responses and viability reductions observed among treatments. Previous studies have demonstrated that fucoxanthin and fucoxanthinol exert variable antiproliferative effects depending on the colorectal cancer cell line evaluated, supporting the concept that tumor heterogeneity strongly conditions treatment sensitivity. Likewise, differences in oxidative stress handling, mitochondrial function, and expression of anti-apoptotic proteins among colorectal cancer cell lines have been associated with variable responses to chemotherapeutic agents and marine-derived compounds. Therefore, the distinct responses observed in Caco-2 and HT-29 cells in the present study may reflect the heterogeneous nature of colorectal tumors and support the potential relevance of personalized adjuvant strategies based on tumor phenotype [34,35,52].

The increased activation of caspases 3/7 observed after treatment with the supplements combined with doxorubicin may be associated with alterations in cellular redox homeostasis and the induction of apoptotic pathways. Several bioactive compounds present in these formulations, including fucoxanthin, polyphenols, and tocotrienols, have been reported to exert dual antioxidant and pro-oxidant effects depending on the cellular redox environment, concentration, and availability of transition metals. Under certain conditions, these compounds may reduce oxidative stress and protect cellular components, whereas in cancer cells they can also promote ROS accumulation and oxidative damage associated with apoptosis induction [28,36,53,54]. Although oxidative stress markers were not directly measured in the present study, the enhanced activation of caspases observed in combined treatments is consistent with previous reports describing apoptosis-promoting effects of these compounds in combination with conventional chemotherapeutic agents [38]. While the present study focused on apoptosis-associated responses through caspase-3/7 activation, several upstream signaling pathways previously reported for the bioactive compounds present in the evaluated formulations may contribute to the biological effects observed herein. In colorectal cancer, the PI3K/Akt/mTOR signaling axis is recognized as one of the major pathways regulating proliferation, survival, metabolism, and resistance to apoptosis, and its dysregulation is frequently associated with tumor progression and poor therapeutic response. Recent reviews have highlighted the importance of this pathway as a therapeutic target in colorectal cancer and have documented the ability of numerous natural products to modulate its activity [55,56]. Fucoxanthin, one of the principal marine carotenoids present in brown algae, has been reported to induce apoptosis, inhibit proliferation, and interfere with PI3K/Akt-associated signaling in several cancer models, while fucoidan has been associated with suppression of PI3K/Akt/mTOR activity, cell-cycle arrest, and activation of apoptotic pathways [57]. Likewise, tocotrienols and polyphenolic compounds have been shown to regulate survival-related signaling pathways, including PI3K/Akt, NF-κB, and Bcl-2 family proteins, thereby promoting apoptotic responses in colorectal cancer cells.

In addition to signaling regulation, oxidative stress and mitochondrial dysfunction have been proposed as important mechanisms underlying the anticancer activity of several plant- and algae-derived bioactive compounds. Previous studies have demonstrated that fucoxanthin, fucoidan, tocotrienols, astaxanthin, and diverse polyphenols can alter intracellular redox homeostasis, modulate reactive oxygen species (ROS) production, affect mitochondrial membrane integrity, and promote the activation of caspase-dependent apoptotic pathways [58,59]. Although ROS generation, mitochondrial membrane potential, PI3K/Akt signaling, and Bcl-2 family proteins were not directly evaluated in the present study, the increased caspase-3/7 activation observed under combined treatment conditions is consistent with apoptosis-associated mechanisms previously described for these bioactive compounds. Therefore, future studies should investigate these upstream molecular events to better characterize the mechanisms underlying the enhanced apoptotic responses observed in colorectal cancer cells.

The effects observed in spheroid cultures are particularly relevant because three-dimensional models better reproduce several structural and physiological characteristics of solid tumors than conventional monolayer cultures [60]. Tumor spheroids develop gradients of oxygen, nutrients, pH, and proliferation that resemble the in vivo tumor microenvironment and often exhibit increased resistance to chemotherapy due to limited drug penetration and altered cellular interactions [61]. While control spheroids exhibited continuous growth, the combination treatments (supplements 2.1 + 10.0) successfully impaired tumor-like expansion, achieving inhibition comparable to DOX alone. In this context, the ability of the evaluated formulations to reduce spheroid growth suggests that their biological activity is maintained even under more physiologically relevant conditions. The AO/EB staining performed in spheroid cultures was intended as a qualitative visualization tool to identify cell death-associated morphological patterns within the three-dimensional structures. Although increased apoptotic fluorescence signals were observed in several treatment groups, these observations should be interpreted as qualitative evidence supporting the viability, caspase-3/7 activation, and spheroid growth inhibition results rather than as a quantitative measurement of apoptotic or necrotic cell populations. Previous studies have shown that astaxanthin and other polyphenol-rich mixtures effectively downregulated Wnt/β-catenin and other tumor-progression pathways, suggesting that these natural agents could be valuable in modulating the complex tumor microenvironment [62]. Similarly, fucoxanthin and fucoxanthinol have retained antiproliferative activity in colorectal cancer models, including experimental conditions that better resemble in vivo tumor architecture, where they were associated with apoptosis induction and suppression of tumor growth [52]. Furthermore, the use of 3D models has become increasingly important for evaluating the translational potential of anticancer compounds because many agents showing promising activity in 2D cultures fail to maintain efficacy in spheroids or animal models. Therefore, the inhibition of spheroid growth observed in the present study strengthens the potential relevance of these formulations as experimental adjuvant candidates in colorectal cancer therapy.

Although the present study demonstrated enhanced antitumor-associated effects in vitro when formulations 2.1 or 10.0 were combined with DOX, these findings should be interpreted as proof-of-concept evidence obtained using a reference cytotoxic agent. Further validation using chemotherapeutic agents more commonly employed in colorectal cancer treatment, such as 5-fluorouracil, oxaliplatin, and irinotecan, will be necessary to determine the translational relevance of these formulations in clinically representative therapeutic schemes. Nevertheless, the present study did not directly evaluate reduced DOX concentrations or protection against toxicity in non-tumor models under combined treatment conditions. Furthermore, all combination treatments were evaluated using fixed concentrations corresponding to the IC_50_ values of the supplements. Therefore, future studies should investigate dose-reduction strategies using multiple concentration combinations to determine whether comparable anticancer effects can be achieved while potentially minimizing chemotherapy-associated toxicity. Accordingly, the comparable inhibitory effect observed for the 2.1 + 10.0 combination in HT-29 spheroids should be interpreted as preliminary evidence supporting the biological activity of mixed natural formulations in a 3D tumor-like model, rather than as proof of DOX dose reduction. Doxorubicin remains an effective anticancer agent; however, its clinical use is limited by important adverse effects, including cardiotoxicity, oxidative damage, and systemic toxicity. Similar strategies combining natural bioactive compounds with conventional chemotherapy have shown promising results in preclinical models [63]. For instance, fucoidan has been reported to enhance the antitumor activity of doxorubicin while reducing treatment-associated toxicity in murine cancer models, partly through apoptosis modulation and immune-related mechanisms [64,65,66]. Likewise, curcumin and resveratrol have been reported to enhance antitumor responses when administered in combination with doxorubicin or cisplatin in animal models, allowing effective tumor suppression at lower drug doses while reducing systemic toxicity [67,68,69]. Also, fucoxanthin increased the cytotoxicity of cisplatin in pancreatic cancer, inhibiting cell proliferation, inducing cell death, switching mitochondrial respiration to aerobic glycolysis, and decreasing whole-cell ATP levels or strongly attenuating the anti-proliferative effect of 5-FU chemotherapy [70,71,72]. These findings support the concept that natural supplements may function as adjuvant agents capable of improving the therapeutic performance of conventional anticancer drugs. Although the present study demonstrated enhanced antitumor effects in vitro when 2.1 or 10.0 supplements were combined with doxorubicin, further validation in murine colorectal cancer models will be necessary to determine whether these combinations can maintain therapeutic efficacy while potentially reducing chemotherapy-associated toxicity. However, these observations should be interpreted cautiously, since formulation 10.0 retained measurable activity in CCD-841 cells and the combination treatments were not evaluated in non-tumorigenic models. Furthermore, future studies should assess these formulations in combination with chemotherapeutic agents more commonly used in colorectal cancer treatment, such as 5-fluorouracil and oxaliplatin, while also evaluating their selectivity and safety in non-tumorigenic cells to better determine their translational relevance in clinically representative therapeutic schemes. Finally, the mechanistic interpretation of the present findings is limited by the lack of direct measurements of oxidative stress-related biomarkers. Therefore, the involvement of redox-regulatory mechanisms remains speculative and should be addressed in future studies through the evaluation of ROS production, mitochondrial function, and antioxidant responses.

## 4. Materials and Methods

### 4.1. Dietary Supplements and Reagents

The dietary supplements were purchased from BioMaussan^®^ (Mexico City, Mexico) and contain the following formulation per 100 mL, according to their declared labeling: 2.0 algas marinas (astaxanthin 20 mg, fucoxanthin 6.6 mg, apple polyphenols 46 mg), 2.1 algas marinas premium (astaxanthin and fucoxanthin 1950 mg and apple polyphenols 1250 mg), 3.0 astaxantina formula especial (astaxanthin 5000 mg), 4.0 fucoxantina formula especial (fucoxanthin 3330 mg), and 10.0 ultra (fucoidan 7142 mg and tocotrienols 6550 mg). The chemical composition of the evaluated dietary supplements was previously characterized by high-performance liquid chromatography–mass spectrometry (HPLC-MS), confirming the presence of the principal bioactive compounds declared by the manufacturer, including fucoxanthin, fucoidan, tocotrienols, astaxanthin, and polyphenols [15]. Doxorubicin hydrochloride (DOX; Zytokil^®^, Laboratorios PISA, Mexico City, Mexico) was used as a reference chemotherapeutic agent and positive control. Stock solutions were prepared in sterile conditions according to the manufacturers’ specifications and diluted in culture medium immediately before use. Final solvent concentrations never exceeded 0.1% (*v*/*v*).

### 4.2. Cell Lines and Cell Culture Conditions

Human colorectal cancer cell lines HT-29 (HTB-38) and Caco2 (HTB-37), ovarian cancer cells OVCAR-3 (HTB-161), prostate cancer cells PC-3 (CRL-1435), and the normal human colon epithelial cell line CCD 841 CoN (CRL-1790) were obtained from the American Type Culture Collection (ATCC, Manassas, VA, USA). OVCAR-3 cells were cultured in RPMI-1640 (Gibco, Grand Island, NY, USA, Cat. 22400-089) supplemented with 10% (*v*/*v*) FBS. PC-3, HT-29, Caco2 and normal colon CCD 841 cells were maintained in DMEM (Gibco, Grand Island, NY, USA, Cat. 11995-065) supplemented with 10% (*v*/*v*) FBS (FBS, Gibco, Grand Island, NY, USA, Cat. 26140-079). Cells were incubated at 37 °C in a humidified atmosphere containing 5% CO_2_. Cells were subcultured at 70–80% confluence.

### 4.3. Cytotoxicity Assay, IC_50_ Determination and Selectivity Index (SI)

Cell viability was assessed using the MTT assay [73] (Bio basic Canada Inc., Markham, ON, Canada, Cat. T0793). Due to differences in growth rate and metabolic activity among cell lines, each cell type was seeded at a specific density in 96-well plates containing 200 µL of complete culture medium per well. The seeding densities were as follows: OVCAR-3 (30,000 cells/well), PC-3 (5000 cells/well), Caco-2 (2000 cells/well), HT-29 (6000 cells/well), and CCD 841 CoN (10,000 cells/well). After seeding, cells were allowed to adhere for 24 h prior to treatment. BioMaussan^®^ dietary supplements and doxorubicin were tested individually over a range of concentrations for 24 h. After incubation, MTT solution was added and plates were incubated for an additional 4 h. Formazan crystals were solubilized with 100 µL of dimethyl sulfoxide (DMSO; Thermo Fisher Scientific, Waltham, MA, USA; Cat. D128-1), and absorbance was measured at 570 nm using a microplate spectrophotometer (synergy™ 2, BioTek Instruments, Winooski, VT, USA). IC_50_ values were calculated using nonlinear regression analysis using GraphPad Prism software ver 8.0.1 [74]. All experiments were performed in triplicate and repeated in three independent assays. The IC_50_ values obtained for each supplement and for DOX were subsequently used as the working concentrations in all downstream experiments. To provide a preliminary estimation of tumor selectivity, the Selectivity Index (SI) was calculated using the IC_50_ value obtained in the non-tumorigenic colon epithelial cell line CCD-481 divided by the IC_50_ value obtained in each colorectal cancer cell line. The following formula was used: SI = IC_50_ CCD − 481/IC_50_ tumor cell line [75]. SI values greater than 1 indicate preferential cytotoxicity toward tumor cells, whereas higher SI values suggest greater selectivity. This approach has been widely used in in vitro cytotoxicity studies to estimate the relative selectivity of compounds or extracts toward cancer cells compared with non-tumorigenic reference cells [75].

### 4.4. Apoptosis Analysis by AO/EB Staining in 3D Spheroids

Apoptotic and necrotic cell populations in HT-29 and Caco2 spheroids were evaluated using acridine orange/ethidium bromide (AO/EB) double staining [76]. The staining was performed only on day 5 after treatment with dietary supplements and DOX at their corresponding IC_50_ concentrations.

Spheroids were incubated with a fluorescent staining solution containing 100 µg/mL acridine orange (AO) and 100 µg/mL ethidium bromide (EB) (AO/EB; Sigma-Aldrich, St. Louis, MO, USA; Cat. 318337 and E7637). A volume of 8 µL of the staining mixture was added to each well. Following staining, spheroids were immediately visualized using an EVOS M3000 inverted fluorescence microscope (Thermo Fisher Scientific, Waltham, MA, USA) at 10× magnification. Imaging was performed using the built-in green and Texas Red light cubes of the system. Cells in spheroids were classified according to nuclear morphology and fluorescence emission as follows: viable cells (green fluorescence with intact nuclear morphology), early apoptotic cells (yellow-green fluorescence with chromatin condensation), late apoptotic cells (orange fluorescence with condensed or fragmented nuclei), and necrotic cells (intense red fluorescence). The AO/EB staining assay was performed in triplicate.

### 4.5. Caspase-3/7 Activity Assay

HT-29 and Caco2 cells were seeded at a density of 8 × 10^3^ cells/well and allowed to attach overnight. Cells were subsequently exposed to doxorubicin (DOX) and the dietary supplements, either individually or in combination, at their corresponding IC_50_ concentrations for 4 h.

Caspase-3/7 activation was evaluated using the CellEvent™ Caspase-3/7 Green ReadyProbes™ Reagent (Invitrogen, Carlsbad, CA, USA; Cat. R37111), following the manufacturer’s recommendations. After incubation with the probe, fluorescence was assessed using an EVOS™ M3000 inverted fluorescence imaging system (Thermo Fisher Scientific, Waltham, MA, USA). Cells were visualized at 10× magnification using the Texas Red light cube of the instrument. The number of caspase-positive cells was determined by fluorescence signal detection. All experiments were independently repeated three times, each including three technical replicates. The inclusion of formulation 10.0 and DOX as single-treatment controls was necessary to establish appropriate experimental references for comparison with the novel combination treatments evaluated in the present study.

### 4.6. Spheroid Culture

Three-dimensional spheroids from the HT-29 and Caco2 colorectal cancer cell lines were generated using Corning^®^ Spheroid Microplates (Corning, NY, USA, Cat. CLS4520) following the manufacturer’s instructions [77]. Briefly, cells were seeded into the ultra-low attachment round-bottom wells of the spheroid microplates at densities of 1000 cells per well for Caco2 and 2000 cells per well for HT-29 to promote uniform spheroid formation. Plates were incubated under standard culture conditions (37 °C, 5% CO_2_), allowing spontaneous cell aggregation and compaction into multicellular spheroids. Culture medium was replenished on day 4. Spheroids were allowed to form and stabilize for 4 days prior to the addition of treatments and subsequent analyses.

### 4.7. Spheroid Treatments

Once compact and morphologically stable spheroids were formed (day 4), HT-29 and Caco2 were subjected to treatment with the plant- and algae-derived supplements (formulations 2.1 and 10.0), doxorubicin (DOX), and their corresponding combinations. Treatments were prepared in complete culture medium and added directly to each well without disrupting spheroid integrity. The evaluated conditions included individual treatments (2.1, 10.0, DOX), dual combinations (DOX + 2.1 and DOX + 10.0), and the supplement-only combination (2.1 + 10.0). Untreated spheroids served as controls.

Following treatment, spheroids were maintained under standard incubation conditions (37 °C, 5% CO_2_) for up to five additional days.

### 4.8. Spheroid Diameter Measurement

Spheroid growth and structural integrity were monitored over time by measuring spheroid diameter at defined intervals (days 0–5 post-treatment). Phase-contrast images were acquired using an EVOS M3000 inverted microscope (Thermo Fisher Scientific, Waltham, MA, USA) at 10× magnification.

For each spheroid, size determination was performed by averaging three independent measurements: the largest and smallest vertical or horizontal diameters, as well as the diagonal diameter. All measurements were obtained using ImageJ Software ver 1.54p [78]. Measurements were conducted on three spheroids per condition to ensure reproducibility. Changes in spheroid diameter were used as an indicator of treatment-induced effects on three-dimensional tumor growth.

Growth curves (control, single-agent and combination treatments) were generated by plotting the mean spheroid diameter over time, and graphical representations were prepared using GraphPad Prism software (ver 8.0.1).

### 4.9. Statistical Analysis

Data are expressed as mean ± SEM from at least three independent experiments. Statistical significance was determined using one-way or two-way ANOVA followed by appropriate post hoc tests with GraphPad Prism software ver 8.0.1. Differences were considered statistically significant at *p* < 0.05.

## 5. Conclusions

The present study demonstrates that the plant- and algae-derived supplements 2.1 and 10.0 possess biologically relevant antitumoral activity in colorectal cancer models and can enhance the anticancer effects of doxorubicin through apoptosis-associated mechanisms. Among the evaluated supplements, formulation 10.0 exhibited the strongest intrinsic cytotoxicity. Importantly, combined treatments with doxorubicin markedly reduced cell viability in both Caco2 and HT-29 cells and significantly increased caspase-3/7 activation, especially in the 10.0 + DOX treatment. Furthermore, the antitumoral effects were maintained in three-dimensional spheroid models, where the combination of formulations 2.1 + 10.0 inhibited HT-29 spheroid growth by approximately 30–35%, producing effects comparable to doxorubicin alone while also promoting apoptotic features. Qualitative AO/EB staining observations in three-dimensional spheroids were consistent with the reduced spheroid growth and increased caspase-3/7 activation observed following treatment, supporting the presence of apoptosis-associated cellular responses. However, additional studies employing dedicated quantitative apoptosis assays will be necessary to further characterize the extent and dynamics of cell death induced by these formulations.

These findings support the hypothesis that formulations enriched in fucoxanthin, fucoidan, tocotrienols, astaxanthin, and polyphenols may modulate multiple cellular pathways associated with colorectal cancer progression, including apoptosis and tumor growth. From a translational perspective, supplements 2.1 and 10.0 emerge as promising candidates for development as adjuvant agents in colorectal cancer chemotherapy, particularly in combination with conventional drugs. Their ability to enhance antitumoral responses in both 2D and 3D models suggests that these formulations may contribute to improving therapeutic efficacy while potentially reducing the amount of chemotherapeutic drug required to achieve biological effects.

## Figures and Tables

**Table 1:** IC_50_ values (μg/mL) of dietary supplements on ovary, prostate and colon cell lines.

	Ovary (OVCAR3)	Prostate (PC3)	Colon (Caco2)	Colon (HT-29)	Colon (CCD-481)
2.0	>1000 ^a^	>1000 ^a^	>1000 ^a^	>1000 ^a^	>1000 ^a^
2.1	>1000 ^a^	>1000 ^a^	340.5 ± 59.13 ^b^	90.99 ± 28.09 ^b^	>1000 ^a^
3.0	>1000 ^a^	>1000 ^a^	>1000 ^a^	>1000 ^a^	>1000 ^a^
4.0	>1000 ^a^	>1000 ^a^	>1000 ^a^	>1000 ^a^	>1000 ^a^
10.0	783.7 ± 58.43 ^b^	156.5 ± 34.5 ^b^	158.70 ± 17.79 ^c^	28.6 ± 6.08 ^c^	40.98 ± 4.6 ^b^
DOX	7.53 ± 1.63 ^c^	16.22 ± 1.83 ^c^	6.50 ± 0.29 ^d^	9.51 ± 0.92 ^c^	39.3 ± 4.1 ^b^

IC_50_ values of dietary supplements (2.0, 2.1, 3.0, 4.0, and 10.0) and doxorubicin (DOX) after 24 h treatment in cancer and normal colon (CCD-481) cell lines. Values represent mean ± SEM (*n* = 3) determined by MTT assay. Different superscript letters within the same column indicate statistically significant differences among treatments, as determined by one-way ANOVA followed by Tukey’s multiple-comparison test (*p* < 0.05). The superscript letters (a, b, c, d) are used solely as statistical grouping labels and do not represent different *p*-value thresholds; all comparisons were evaluated using the same significance level (*p* < 0.05). Values sharing at least one common superscript letter are not significantly different, whereas values with different superscript letters differ significantly (*p* < 0.05).

## Data Availability

Dataset available on request from the authors due to privacy restrictions.
